# The Effect of Adaptation Training on Controlling Maladaptation Behaviors in Adolescents with Asthma Based on Roy Adaptation Model

**Published:** 2018-02

**Authors:** Nasrollah Alimohammadi, Bibi Maleki, Samira Abbasi, Behzad Shakerian, Zeinab Hemati

**Affiliations:** 1 Department of Critical Care, Faculty of Nursing and Midwifery, Isfahan University of Medical Sciences, Isfahan, Iran; 2 Faculty of Nursing and Midwifery, Isfahan University of Medical Sciences, Isfahan, Iran; 3 Department of Psychiatric Nursing, Faculty of Nursing and Midwifery, Isfahan University of Medical Sciences, Isfahan, Iran; 4 Subspecialty of Allergy and Immunology, Shariati Hospital, Isfahan, Iran; 5 Nursing and Midwifery Care Research Center, Faculty of Nursing and Midwifery, Isfahan University of Medical Sciences, Isfahan, Iran.

**Keywords:** Asthma, Adolescents, Maladaptation Behaviors, Roy Adaptation Model

## Abstract

**Background:**

Asthma is a common long term inflammatory disease during adolescent. Absence of school education and reduction of mental and social mindedness are among the most common problems found in adolescents with asthma. Therefore, the present study was aimed to examine the effect of Roy adaptation model on controlling maladaptation behaviors in adolescents with asthma.

**Materials and Methods:**

This study is a semi-experimental research that was conducted with the participation of all adolescents with asthma referred to the Asthma and Allergy Clinic of Shariati Hospital. Random sampling was used for a total of 64 adolescents to have two groups of intervention and control. Data collection was through a questionnaire based on the Roy’s Adaptation Model. Over six weeks, adolescents were trained in six two-hour sessions. Data were analyzed by descriptive and analytical statistics consisting of Mann-Whitney, ANOVA, paired t-test and independent t-test.

**Results:**

The mean age of adolescents with asthma in the intervention and control groups was 15.8±3.5 and 14.8±3.5 years, respectively. Also, the mean score of maladaptation behaviors in four physiological, self-concept, role-function and interdependence modes had a significant difference in intervention group before and after training (p<0.001), but there was no significant difference in all modes before and after intervention in control group (p>0.05).

**Conclusion:**

Given the effect of Roy adaptation model on the different aspects of maladaptation behaviors in adolescents with asthma, it is recommended to use this model as a healthcare intervention for controlling maladaptation behaviors in adolescents with other chronic disease.

## INTRODUCTION

Asthma is one of the most common chronic diseases among children and adolescents worldwide, and the foremost cause for inability in children (1). As shown, 1.6 million emergency visits, more than two million hospitalizations, and around 1.1 million days of school absence, main activity limitation, and disappointing performance in children are nearly due to asthma (2). It is reported that this disease is prevalent in 5–10% of children and adolescents worldwide. The prevalence of asthma in Iran is 2.7–35.4% in children, with the highest related mortality in adolescents of 11–17 years (3). Asthma in adolescence poses several challenges for adolescents and causes to add to the possibility of symptoms’ exacerbation and asthma attacks due to occurrence of important physical, psychological, emotional, sexual, and social variations in adolescence (4).

Co-occurrence of disease complications such as treatment procedures, mental and physical stress, change in roles, interactions and communications in the changes and transformations of adolescence, may prevent an adolescent from having self-control (5). Although much progress has been made in the management of asthma in adolescents, but its complications is increasing (3). The inability to perform optimal ventilation restricts the level of tolerance to physical activity and this can affect social interactions, leading to social isolation of patient (6).

Generally, asthma affects not only the health of children and adolescents, but also has an impact on other aspects of life such as coping style, mental performance and overall quality of life. The results of study showed that asthma not only affects children and adolescents’ physical status but also influences their social, emotional and educational status, since it can lead to school absenteeism, academic failure and the reduction of social and psychological adaptation. Therefore, considering adaptation decrease in physical, social and psychological aspects (7,8), and the failure of individual’s effort to build effective adaptation, effective nursing care gains more importance as nurses play a major role in supporting patients with chronic diseases to have adaptation (9,10).

Adaptation responses reduce the amount of energy needed to deal with situations in patients. Roy adaptation model (11), with the aim of performing nursing care measures to improve adaptation responses in four psychological, self-concept, role-function and independence modes, is among nursing care patterns dealing widely and deeply with the issue of adaptation in patients with chronic diseases (12).

According to this pattern, nurses systematically and accurately deal with the evaluation of patients through interviews, observation, and measurement. Maladaptation behaviors are defined as tendencies or actions that do not allow an individual to adjust well to certain situations. These behaviors are typically disruptive and dysfunctional behaviors which can range from mild to severe in scope, determined in four modes with behavior stimuli, and subsequently the exact caring and educational programs will be designed to meet the maladaptive behaviors (11,13, 14). In this connection, Whittemore study presented that Roy adaptation model which led to increased level of adaptation in self-concept and role-function modes in adolescents with diabetes (15). Furthermore, the study conducted by Afrasiabifar also displayed that Roy model is effective in increasing compliance of hemodialysis patients in two modes of self-concept and physiology (16).

Diagnosing and offering appropriate care solutions that lead to increased adolescents compliance with existing conditions gain importance due to the chronic nature of asthma and the fact that adolescents have long-term use of drugs, and also taking into account patients’ care needs along with the issue that high energy, and power generation are regarded as social values.

Therefore this study was aimed to examine the effect of Roy adaptation model on controlling maladaptation behaviors in adolescents with asthma.

## MATERIALS AND METHODS

This semi-experimental study was conducted from April 2016 to December 2016. The sample size was calculated as 32 in each group based on the data of similar studies: (d=. /7s^2^, α=0.05, β=0.2) (17).

$$\frac{2{\left(z1+z2\right)}^{2}{\text{s}}^{2}}{{\text{d}}^{2}}$$

The study was conducted in Shariati Hospital, Asthma and Allergy Clinic, and sampling was done on the adolescents referring to Shariati Hospital, Isfahan. This means that researchers attended the clinic and convenient sampling was used for selecting the adolescents based upon the inclusion criteria. After explaining the purpose of research and getting informed consent, Roy assessment form was filled out by subjects. For the next stage, random sampling (random number table) was used to put the adolescents in two groups.

According to GINA guidelines, the subspecialist selected the adolescents with moderate to severe asthma and history of one or more asthma attack. The prospective subjects were capable to answer the questions and debate in training classes, were full conscious, were 11–21 years old and had not previously participated in similar training. Furthermore, the following conditions disqualified the subjects from inclusion in the study: absence in two successive and/or one fourth of all the sessions; other long-lasting disease; mental health and mental retardation (by medical file and the physician); history of taking drugs with mental effects (by medical file and the physician); emergency conditions throughout the study; stressful incidence such as bereavement during the study in both groups (based on the patients’ statement before and during the study since they were already informed of the participation in this study); and withdrawal from the study.

To do intervention based on Roy adaptation model, maladaptation behaviors were identified according to the following steps:

In the first step of evaluation, interview and observation were used to determine the maladaptation behaviors related to four modes of adaptation (as an example, reducing the adolescent’s ability to training exercise of daily living as a maladaptive behavior in Physiologic mode). In the second step, three different types of residual, focal and contextual stimuli were identified regarding the stimuli of each maladaptation behavior in four modes. The definition of different types of stimuli are as below; focal stimuli are those which are most immediately confronting the human adaptive system (for example: asthma); contextual stimuli are all other stimuli with effect on the focal stimuli (for example: dyspnea while taking a shower); residual stimuli are environmental factors with unknown effects on the current situation (for example: belief and thought about not being able to take a shower safely).”

In the third step, the nursing diagnoses were listed in detail. In the fourth step, short and long term objectives were designed by the researcher, adolescents and their parents. In the fifth step, the interventions were done according to a training protocol conducted by care provider team.

This means that in the first session, the overall and behavioral objectives, evaluation methods, definition of disease and kinds of asthma, causes of asthma attacks and symptoms were presented. Session two dealt with training of childhood asthma, asthma causes and ways to prevent it. The third session focused on training and exercise-induced asthma, causes and measures to prevent it. In fourth session, strategies to cope with depression and anxiety along with referring to psychiatrist, were employed in some cases to achieve adaptation in different modes. In the fifth session, dietary advice and education on asthma was conducted.

At the end of the program, a comprehensive educational booklet containing content on asthma and treatment and care methods was given to the adolescents that participated in intervention group. Training sessions were conducted by physicians, nurses and psychologists as a group and individually, and were held every week for two hours through lectures, brainstorming and use of teaching aids like PowerPoint. The researcher’s phone number was given to patients to answer their questions and provide guidance and support for them during the two months’ follow-up. Also, to enhance the sense of support in the intervention group the researcher called them once a week (for two months) and answered their questions related to their conditions and care plan method. The sixth session was allocated to filling out the Roy assessment form in two groups after the end of the last training session.

In the present study, the adolescents in the control group received teaching and problem solving by physician in regular visits. Two months later, a questionnaire was filled out by the participants in both groups.

Data collection was done through a questionnaire containing items related to the demographic variables and four adaptation modes. The questionnaire was of a five-item scale type assessing the physiological mode by 26 questions, self-concept mode by 23, role function mode by 12 and interdependence mode 12 questions. The scores of physiological modes ranged (0–104), by means of a five-item scale (never, rarely, sometime, often and always), self-concept mode (0–92), with a five-item scale (always, frequently, sometimes, rarely and never), role function mode (0–48), with a five-item scale (never, shortened period, sometimes, often and always), and interdependence mode (0–48), with a five-item scale (fully disagree, disagree, no opinion, agree, and fully agree). The design of Roy assessment form was based on library resources and internet search and using related tools. This form was distributed among 9 academic members of Isfahan Nursing and Midwifery School and one academic member in the School of Medicine so as to determine face and content validity, and the required adjustments were made based on their suggestions. To determine the reliability of the questionnaire, a pilot study was conducted and Cronbach’s alpha was calculated (0.78). Finally, data were analysed by Chi-square, Mann-Whitney, Independent *t-*test and Paired *t*-test with confidence interval of 95% through SPSS version 19.

## RESULTS

The mean age of adolescents with asthma in the intervention and control groups was 15.8±3.5 and 14.8±3.5 years, respectively. The duration of disease in the intervention group was 5.4±1.9 and 5.3±2.9 in control group and the independent t-test did not show significant differences in these variables between the two groups (p>0.05). Also 51.4% of the subjects in the intervention group and 53.3% in the control group were female, and the education level of 42.9% of the subjects in intervention group and 36.7% of them in the control group was high school. Comparison of individual characteristics of parents showed that there was no statistically significant difference between the two groups in terms of father and mother’s occupation and their education level (p>0.05) (Table 1).

**Table 1.** Demographic characteristics of asthma patients in the two groups

**Variables**	**Intervention Group**	**Control Group**	**P value**
Mean age (yrs.) (Mean±SD)	15.8±3.01	14.8±3.5	0.21
Sex N (%)			
Male	17(48.6)	14(46.7)	0.88
Female	18(51.4)	16(53.3)	
Education level N (%)			
Elementary	2(5.7)	5(16.7)	
Secondary	11(31.4)	10(33.3)	0.19
Diploma	15(42.9)	11(36.7)	
University degree	7(20)	4(13.3)	
Duration of asthma (yrs.) (Mean±SD)	5.4±1.9	5.3±2.9	0.79

Also, the mean score of maladaptation behaviors in four physiological, self-concept, role-function and interdependence modes had a significant difference in intervention group before and after training (p<0.001). This means that the decline in mean scores of maladaptation behaviors after training was related to role-function mode and then interdependence mode. On the contrast, there was no significant difference in all modes before and after training in control group (p>0.05) (Tables 2, 3).

**Table 2.** Comparison of mean maladaptation behavior in intervention group before and after training in four modes

**Domains**	**Time**	**Before**	**After**	**Paired t- test results**

		**(M±SD)**	**(M±SD)**	
Physiologic		49.3 ±10.1	41.4 ±6.4	P<0.001
Self-concept		44.7 ±9.1	36.4 ±6.6	P<0.001
Role-function		18.8 ±5.9	12.8 ±5.9	P<0.001
Interdependence		23.7 ±5.5	16.5 ±6.2	P<0.001
Total Score for Maladaptation Behaviors		136.4 ±26.1	107.3 ±18.7	P<0.001

**Table 3.** Comparison of mean maladaptation behavior in two groups after training in four modes

**Domains**	**Time**	**Intervention**	**Control**	**Independent t-test results**

		**(M±SD)**	**(M±SD)**	
Physiologic		41.4 ±6.4	49.8 ±6.1	P<0.001
Self-concept		36.4 ±6.6	44.8 ±21.5	P=0.04
Role-function		12.8 ±5.9	17.1 ±8.9	P=0.03
Interdependence		16.5 ±6.2	22.4 ±8.5	P=0.003
Total Score for Maladaptation Behaviors		107.3 ±18.7	134.1 ±34.2	P<0.001

## DISCUSSION

The present study aimed to investigate the effect of adaptation training on controlling maladaptation behaviors in adolescents with asthma based on Roy adaptation model. The results showed a significant difference in using this model for different aspects of maladaptation behaviors (physiological, self-concept, role-function and interdependence) in adolescents with asthma in the intervention group. In physiological mode, the results of Srof et al. showed that adolescents’ participation in training sessions of coping skills within two months resulted in improving their quality of life in the mode of signs and symptoms of disease and activity limitation (18). In another study, adolescents’ participation in asthma self-management education program led them to get increased knowledge about the disease and consequently resulted in increasing their knowledge of medical treatment (2).

Considering the results of the study, holding group meetings with intraprofessional cooperation with nurses, physicians, psychologists and paying attention to the needs of adolescents before the training, and physiological changes during the training were evident. Moreover, the results showed a significant difference in self-concept and role-function mode in adolescents with asthma in the intervention group. The results of several studies are consistent with the results of the present research in terms of self-concept and role-function modes. For example, the results of the Sharifi and Kaveh study showed that adolescent’s participation in 10 sessions of 90 minutes on training adaptation methods with concepts such as how to express emotions, self-control, time management and adaptation led to decrease in depression and anxiety, increase in social performance and a sense of control over one’s disease (19).

One study suggested that, coping mediates the effect of symptoms of asthma on quality of life among patients (20). Also, Akyil and Erguney examined the training program based on Roy adaptation model considering the adaptation of patients with chronic lung disease. They indicated that training based on this pattern leads significant difference in the number of maladaptation behaviors in self-concept and role-function modes (13).

To explain the obtained results, we can state that the patient’s ability to perform daily activities is one of the most important indicators to assess the adaptation in role-function mode. The findings of this research indicate the effectiveness of the proposed care plan in improving the ability of adolescents with asthma in performing daily life activities. On the other hand, the ultimate goal in chronic diseases is to control the disease by the patient and prevent complications that can be achieved with adaptation behavior (21).

The results showed a significant difference in interdependence mode in adolescents with asthma in the intervention group compared to control group. Grey et al showed that participation of adolescents with diabetes type 1 in six weekly sessions of 90 minutes on adaptation skills lead to decrease in disease impacts and less parent control and finally the independence of adolescent (22). In Akyil and Erguney study, Roy adaptation model did not lead to a significant difference in interdependence mode of families of patients with chronic lung diseases, despite its impact on other adaptation modes (13). Since an important part of the care of chronic patients is done at home and within the family environment, family can have a significant role in proper control of the disease and its symptoms. One of the features of this study was the family involvement in training sessions; therefore, the majority of the adolescents participated in these sessions with their parents (23).

The results of Van De Ven et al. indicated that ineffective adaptation of adolescents against asthma leads to hiding and ignoring the disease and so adolescents mostly employ preventive adaptation method in disease condition. However, patient education based on Roy adaptation model can result in improving self-concept, supporting self-care behaviors and assisting to achieve the skills in order to adapt to chronic conditions of the disease (20).

### Study limitations

The researcher held the sessions at the end of the week since the training sessions overlapped with school timing of the adolescents. This can be seen as the most considerable limitation of the study.

## CONCLUSION

According to the results and impact of Roy adaptation model on different aspects of maladaptation behaviors in adolescents with asthma and also considering the feasibility of the care plan as a noninvasive, non-pharmacological and low cost program in controlling physical and psychological problems by nurses, this model seems to be of beneficial effects in terms of better control of chronic diseases’ conditions for the adolescents.
